# Modeling the seasonal and climate-dependent dynamics of visceral leishmaniasis in Brazil: Implications for transmission and Control

**DOI:** 10.1016/j.idm.2025.11.009

**Published:** 2025-11-25

**Authors:** Quinn H. Adams, Davidson H. Hamer, Lucy R. Hutyra, Gregory A. Wellenius, Kayoko Shioda

**Affiliations:** a Center for Climate and Health, Boston University School of Public Health, Boston, MA, United States of America; b Department of Environmental Health, Boston University School of Public Health, Boston, MA, United States of America; c Department of Global Health, Boston University School of Public Health, Boston, MA, United States of America; d Center on Emerging Infectious Diseases, Boston University, Boston, MA, United States of America; e Section of Infectious Diseases, Department of Medicine, Boston University Chobanian & Avedisian School of Medicine, United States of America; f Department of Earth and Environment, Boston University, Boston, MA, United States of America

**Keywords:** Mathematical modeling, Visceral leishmaniasis, Climate variability, Public health intervention

## Abstract

**Background:**

Visceral leishmaniasis (VL) is a parasitic, zoonotic neglected tropical disease that remains a persistent public health challenge in endemic regions of Brazil, including the state of Maranhão. Transmission dynamics are complex, involving interactions between *Lutzomyia longipalpis* sandflies, canine reservoirs, and human hosts, and are influenced by environmental and climatic variability. Mathematical models are critical tools for understanding these dynamics and identifying opportunities to effectively disrupt transmission.

**Methods:**

Our objective was to develop and calibrate a climate-informed mechanistic model of VL transmission in Maranhão, Brazil, and to evaluate the potential impacts of vector, environmental, and reservoir-targeted interventions. The model incorporates seasonally varying sandfly biting rates and vector recruitment and explicitly accounts for climate variability through the El Niño-Southern Oscillation (ENSO). Transmission rates between populations (human, canine reservoir, and sandfly vector) were calibrated using monthly reported human VL cases from 2007 to 2019 in Maranhão. We simulated the impact of four potential interventions on VL incidence: increased vector mortality, environmental sanitation (reducing vector maturation), expanded canine treatment, and increased canine culling.

**Results:**

The model accurately reproduced the observed temporal trends in monthly human VL cases in Maranhão and quantified the nonlinear effects of potential interventions. Vector control was the most effective standalone strategy, with a 10 % increase in sandfly mortality reducing human cases by 43 %, and a 90 % increase leading to a 96 % decline. Environmental sanitation was similarly impactful, with a 50 % reduction in sandfly maturation lowering cases by 72 %, and a 90 % reduction leading to a 97 % decline. Canine-focused strategies were less effective: expanded canine treatment reduced human cases only up to 69 %, while increased euthanasia had only modest effects. A combined intervention strategy was more effective than any individual measure, reducing cases by 61 % at just a 10 % increase in coverage and achieving substantially greater declines at higher levels.

**Conclusions:**

Climate variability and seasonal dynamics were key drivers of VL transmission in this setting. Our findings highlight the importance of integrating vector control and environmental management as core components of VL mitigation strategies. While canine-focused interventions may contribute incremental benefits, they are less effective than other interventions and are insufficient when implemented in isolation.

## 1 Introduction

Visceral leishmaniasis (VL), a neglected tropical disease caused by *Leishmania* parasites and transmitted by *Lutzomyia* sandflies, remains a persistent public health challenge in endemic regions (Burza et al., 2018). In Brazil, where VL is endemic, domestic dogs serve as key reservoirs, amplifying transmission in urban and peri-urban areas (Courtenay et al., 2002). The distribution and abundance of sandfly populations are strongly influenced by environmental factors, such as temperature, humidity, and rainfall(Q. H. Adams, Milando, et al., 2025); however, the precise mechanisms by which seasonality modulates the transmission of VL remain poorly characterized (Rock et al., 2016). These seasonal patterns are also inconsistent across different regions(Q. Adams, Milando, et al., 2025). For example, in the Northeast region of Brazil, sandfly populations generally peak toward the end of the rainy season. In contrast, in the Amazon region, populations peak at the end of the dry season (Rock et al., 2016). Given the complex interplay between vector abundance, host reservoirs, and environmental conditions, mathematical models can capture seasonal transmission dynamics and evaluate the effectiveness of different intervention strategies.

Maranhão, Brazil, bears one of the highest burdens of VL across the country. Located in the northeastern region, Maranhão experiences two distinct seasons: a rainy season from December to May and a dry season from June to November. Its tropical climate, characterized by warm and humid conditions, provides an optimal environment for the development and survival of *Lutzomyia longipalpis,* the primary vector of VL in Brazil (da Saúde www.saude.gov.br/bvs,n.d.). In addition to its intrinsic seasonal variations, Maranhão's precipitation patterns are significantly influenced by the El Niño Southern Oscillation (ENSO) (Hastenrath, 2006). El Niño events tend to suppress rainfall, leading to drier conditions, whereas La Niña events enhance precipitation, creating wetter-than-normal conditions (Correia Filho et al., 2019). These fluctuations in precipitation may impact sandfly population dynamics and, consequently, VL transmission risk(Gutiérrez et al., 2024).

Despite ongoing control efforts through the Visceral Leishmaniasis Control Program (Programa de Vigilância e Controle da Leishmaniose Visceral (PVC-VL)), including robust epidemiologic surveillance, residual insecticide spraying, environmental sanitation efforts, and dissemination of insecticide-treated dog collars, for example, VL transmission remains difficult to control (Costa et al., 2013). The PVC-VL also recommends canine euthanasia as a control strategy; however, its effectiveness in interrupting transmission remains uncertain (França-Silva et al., 2023), and growing ethical and logistical concerns continue to challenge its implementation. Process-based, multi-compartmental models provide a robust framework for disentangling the internal drivers of disease transmission and elucidating the complex mechanisms underlying VL persistence. These mechanistic models, which simulate biological and ecological processes influencing disease spread, are widely used to evaluate the effectiveness of preventive measures and forecast disease dynamics (Ye et al., 2025; de Wit et al., 2024; Procopio et al., 2023; Lambert et al., 2023).

Only a few models of zoonotic VL transmission exist (Buckingham-Jeffery et al., 2019; Modu et al., 2024; Sevá et al., 2016; Shimozako et al., 2017; Zhao et al., 2016), and they vary in their geographic scope, biological details and model structures. To our knowledge, there are no existing models that incorporate correlates of climate variability, particularly ENSO-driven fluctuations in rainfall and temperature, despite their influence on sandfly dynamics in Brazil (Rebêlo et al., 2024). Existing models also rarely focus on Maranhão, one of the country's highest-burden states, and often simplify or leave out key seasonal processes such as vector biting rates or sandfly maturation cycles. Moreover, few prior studies have evaluated multiple intervention strategies within a climate-sensitive modeling framework. Our study addresses these gaps by integrating ENSO-dependent vector traits, seasonal forcing of vector biting rates and recruitment, and region-specific dynamics to better characterize VL transmission in Maranhão. By doing so, we aim to establish a baseline scenario that can inform the evaluation and comparison of public health interventions, ultimately contributing to more effective disease control strategies.

## 2 Methods

### 2.1 Transmission model

We developed a compartmental model to simulate VL transmission dynamics among *Lutzomyia longipalpis* sandflies (vector), domestic dogs (reservoir hosts), and humans. The model extends the classic Susceptible-Exposed-Infected-Recovered (SEIR) framework to explicitly account for vector-host interactions and climate-driven transmission dynamics.

Key assumptions from our model include.

● Humans are dead-end hosts (they acquire disease but do not contribute to onward transmission).
● Recovery confers permanent immunity (Rock et al., 2016).
● The canine population remains stable over time. This assumption is consistent with prior VL modeling studies that treat canine populations as approximately stationary over multi-year periods (Sevá et al., 2016; Buckingham-Jeffery et al., 2019; Martins et al., 2025).

**Fig. 1:**
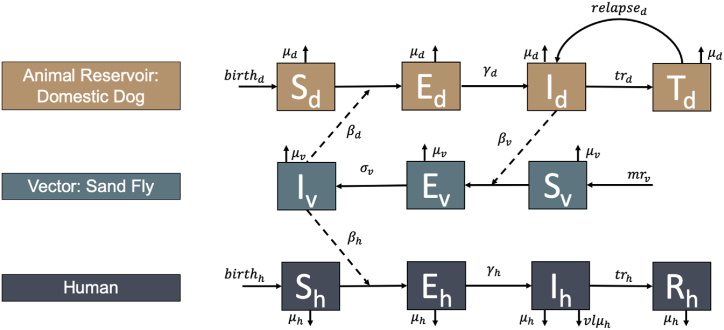
Compartmental model of visceral leishmaniasis (VL) transmission involving domestic dogs (brown), sand fly vectors (blue), and humans (dark blue). The model includes susceptible (S), exposed (E), infectious (I), treated or recovered (T/R) compartments for each population. Solid arrows represent transitions between disease states, while dashed arrows indicate transmission pathways between species. Model equations are given in Equations Equation 1, Equation 2, Equation 3, Equation 4, Equation 5, Equation 6, Equation 7, Equation 8, Equation 9, Equation 10, Equation 11) and parameters are given in Table 1.

The model structure shown in Fig. 1 corresponds to the system of differential equations provided in Equations Equation 1, Equation 2, Equation 3, Equation 4, Equation 5, Equation 6, Equation 7, Equation 8, Equation 9, Equation 10). Definitions and values of parameters in Equations Equation 1, Equation 2, Equation 3, Equation 4, Equation 5, Equation 6, Equation 7, Equation 8, Equation 9, Equation 10) are described in Table 1. Biological parameters were sourced from published literature (Table 1).

**Table 1:** Parameters, definitions, values, and source.

Parameter	Definition	Value	Source
${birth}_{h}$	The birth rate for humans	4.11∗10^-5	World Bank Open Data (Death Rate, Crude (per 1,000 People) - Brazil, n.d.)
${birth}_{d}$	Birth rate for dogs	2.28∗10^-5	Approximated from Junqueira & Galera, 2019 (Junqueira & Galera, 2019)
$\mu_{h}$	Natural death rate for humans	1.64∗10^-5	World Bank Open Data (Birth Rate, Crude (per 1,000 People) - Brazil, n.d.)
${br}_{h}$	Biting rate of sandflies to humans	2.00∗10^-1	Shimozako et al. (2017)
${br}_{d}$	Biting rate of sandflies to dogs	0.333	Buckingham-Jeffery et al. (2019)
$\gamma_{h}$	The inverse of the incubation period	3.78∗10^-4	Pearson et al. (1990)
$\gamma_{d}$	The inverse of the incubation period	0.0111	Pearson et al. (1990)
${tr}_{h}$	Treatment rate among humans	0.5294	Zhao et al. (2016)
${tr}_{d}$	Treatment rate among dogs	0.0233	Zhao et al. (2016)
${vl\mu }_{h}$	VL-specific mortality	6.31∗10^-3	SINAN (Ministry of Health, Brazil)
${mr}_{v}$	Average maturation rate from pupae to adult sandflies	0.0833	Hussaini et al. (2017)
f	proportion female	0.5	Assumed
$n_{d}$	Relative infectiousness of dogs (to sandflies)	0.385	Laurenti et al. (2013)
$\mu_{v}$	Mortality rate of sandflies	0.0714	Hussaini et al. (2017)
${relapse}_{d}$	Relapse rate among dogs	7.083∗10^-3	Hussaini et al. (2017)
*R*	Reporting rate	1/18	Chappuis et al. (2007)

σ_*v*_	Extrinsic incubation period	7 days	Shimozako et al. (2017)

$\beta_{h}$	Human infection rate (sandfly to human)		Estimated

$\beta_{d}$	Canine infection rate (sandfly to dog)		Estimated

$\beta_{v}$	Vector infection rate (dog to sandfly)		Estimated

Human Population:

Equation 1 $$\frac{dS_{h}}{dt}={\text{birth}}_{h}\cdot N_{h}-\frac{\beta_{h}\cdot {\text{br}}_{h}\cdot I_{v}}{N_{h}+N_{d}}\cdot S_{h}-\mu_{h}\cdot S_{h}$$

Equation 2 $$\frac{dE_{h}}{dt}=\frac{\beta_{h}\cdot {\text{br}}_{h}\cdot I_{v}}{N_{h}+N_{d}}\cdot S_{h}-\left(\gamma_{h}+\mu_{h}\right)\gamma_{h}+\mu_{h}\cdot E_{h}$$

Equation 3 $$\frac{dI_{h}}{dt}=\gamma_{h}\cdot E_{h}-\left({\text{tr}}_{h}+\mu_{h}+\text{vl}\mu_{h}\right){\text{tr}}_{h}+\mu_{h}+\text{vl}\mu_{h}\cdot I_{h}$$

Equation 4 $$\frac{dR_{h}}{dt}={\text{tr}}_{h}\cdot I_{h}-\mu_{h}\cdot R_{h}$$

Reservoir Population:

Equation 5 $$\frac{dS_{d}}{dt}={\text{birth}}_{d}\cdot N_{d}-\frac{\beta_{d}\cdot {\text{br}}_{d}\cdot I_{v}}{N_{h}+N_{d}}\cdot S_{d}-\mu_{d}\cdot S_{d}$$

Equation 6 $$\frac{dE_{d}}{dt}=\frac{\beta_{d}\cdot {\text{br}}_{d}\cdot I_{v}}{N_{h}+N_{d}}\cdot S_{d}-\left(\gamma_{d}+\mu_{d}\right)\gamma_{d}+\mu_{d}\cdot E_{d}$$

Equation 7 $$\frac{dI_{d}}{dt}=\gamma_{d}\cdot E_{d}+{\text{relapse}}_{d}\cdot T_{d}-\left({\text{tr}}_{d}+\mu_{d}+\text{vl}\mu_{d}\right){\text{tr}}_{d}+\mu_{d}+\text{vl}\mu_{d}\cdot I_{d}$$

Equation 8 $$\frac{dT_{d}}{dt}={\text{tr}}_{d}\cdot I_{d}-\left({\text{relapse}}_{d}+\mu_{d}\right){\text{relapse}}_{d}+\mu_{d}\cdot T_{d}$$

Vector Population:

Equation 9 $$\frac{dS_{v}}{dt}={\text{mr}}_{v}\cdot f-\frac{\beta_{v}\cdot {\text{br}}_{d}\cdot \left(n_{d}\cdot E_{d}+I_{d}\right)}{N_{h}+N_{d}}\cdot S_{v}-\mu_{v}\cdot S_{v}$$

Equation 10 $$\frac{dE_{v}}{dt}=\frac{\beta_{v}\cdot {br}_{v}\cdot \left(n_{d}\cdot E_{d}+I_{d}\right)}{N_{h}+N_{d}}-\sigma_{v}\cdot S_{v}-\mu_{v}\cdot E_{v}$$

Equation 11 $$\frac{dI_{v}}{dt}=\frac{\beta_{v}\cdot {\text{br}}_{d}\cdot \left(n_{d}\cdot E_{d}+I_{d}\right)}{N_{h}+N_{d}}\cdot S_{v}-\mu_{v}\cdot I_{v}$$

Seasonality and climate variability were incorporated as key ecological drivers of transmission. Seasonal fluctuations were modeled using a flexible two-harmonic Fourier function that modulates sandfly-human biting rate and vector recruitment, two processes known to vary strongly seasonally in Northeastern Brazil. The seasonal multiplier is defined as:

Equation 12 $$f_{seas}\left(t\right)t=1+a_{1}\;\sin\;\left(\frac{2\pi \left(t+\phi \right)}{365.25}\right)+b_{1}\;\cos\;\left(\frac{2\pi \left(t+\phi \right)}{365.25}\right)+a_{2}\;\sin\;\left(\frac{4\pi \left(t+\phi \right)}{365.25}\right)+b_{2}\;\cos\;\left(\frac{4\pi \left(t+\phi \right)}{365.25}\right)$$

Where $a_{1},b_{1}$ are the first-harmonic Fourier coefficients, $a_{2},b_{2}$ are the second-harmonic coefficients, and ϕ is a phase shift (days).

To incorporate interannual climate variability, we allowed ENSO to modify four climate-sensitive vector traits (biting rate amplitude, vector recruitment, extrinsic incubation period, and vector mortality). ENSO anomalies were incorporated using a multiplicative exponential function.

Equation 13 $$X_{ENSO}\left(t\right)=X_{0}\;\cdot \;\exp\left[\left(\alpha \;\cdot \;ENSO\left(t-\tau \right)\right)\right]$$

Where $X_{0}$ represents the baseline value in neutral ENSO conditions, α is a fitted coefficient that quantifies the estimated sensitivity to ENSO, and ENSO(t−τ) represents the ENSO index at a lag of τ months. We used this functional form because additive or linear forms can yield negative or biologically implausible values when anomalies are large. To identify the optimal lag for the ENSO effect on human transmission, we tested a range of ONI lags from 0 to 6 months and selected the lag that minimized the model's negative log-likelihood. The results presented below represent ONI lagged by 4 months. This approach allowed us to capture the delay between climate anomalies and their ecological and epidemiological effects.

## 3 Model parameter estimation

The model was calibrated using monthly VL surveillance data from 2007 through 2019, a 12-year period that reflects real-world reported cases in Maranhão. We ran the model for 57 years so that the simulated VL case counts reached a stable level. To ensure that the model reached an endemic equilibrium before calibration and intervention testing, we simulated a 57-year model initialization period starting in 1950. This approach allowed us to initialize the model under historical El Niño conditions using the available ONI data. By running the model forward for several decades, we allowed the internal dynamics to override arbitrary starting values and stabilize based on the model's structure and long-term climatic forcing. This approach reduced sensitivity to unknown initial conditions and ensured more realistic simulations during the evaluation period. By the time the simulation reached the start of the calibration window in 2007, the model reflected a steady-state baseline appropriate for comparison with real surveillance data and for evaluating the effects of hypothetical interventions.

We calibrated the model using maximum likelihood estimation, assuming a Poisson-distributed likelihood function for observed case counts. Specifically, we minimized the negative log-likelihood by comparing simulated steady-state equilibrium VL incidence to observed surveillance data. The model simulated daily true infections, including both symptomatic and asymptomatic cases, as well as reported cases using the reporting rate constant (*R*). Simulated daily reported cases were then aggregated to the monthly level and were fit to the observed monthly human case count data. The key model parameters estimated included.

● Sandfly-to-canine, canine-to-sandfly, and sandfly-to-human transmission rates (β_d_, β_v_, and β_h_), capturing interspecies interactions (Fig. 1).
● Fourier coefficients and phase shift in vector-to-human biting rates and vector recruitment that are modeled using two-harmonic Fourier functions (Equation (12)).
● Alpha in the ENSO-dependent vector traits (separately for each trait) (Equation (13))

To quantify model uncertainty, we applied parametric bootstrapping to generate confidence intervals around the model fit and evaluations of intervention effectiveness. Specifically, we performed 1000 bootstrap iterations to assess variability in model estimates. Each iteration resampled the observed case data with replacement. The model was refitted to each resampled dataset using the NLL. Point estimates were derived from the median of the bootstrapped parameter estimates, and 95 % confidence intervals represent the 2.5th and 97.5th percentiles of the parameter estimate distribution.

### 3.1 Data sources

We obtained monthly confirmed VL case data from the Brazilian Ministry of Health Information System for Notifiable Diseases (SINAN) (Ministry of Health, Brazil). Due to disruptions in surveillance and reporting following the COVID-19 pandemic (Silveira et al., 2024), we restricted our analysis to 2007 through 2019 to ensure reliable parameterization of the compartmental model.

We used the Oceanic Niño Index (ONI) from the National Oceanic and Atmospheric Administration's Climate Prediction Center (NOAA/PSL Climate Prediction Centern.d.), which is one of several methods for tracking the state of ENSO. The ONI is calculated as the 3-month moving average of sea surface temperature anomalies in the Niño 3.4 region. Values greater than or equal to 0.5 °C indicate El Niño conditions while values less than or equal to −0.5 °C indicate La Niña conditions. The ONI was used to inform a vector biting rate in the transmission model. We simulated human cases using ONI data from 1950 through 2019. This ensured that the simulated disease dynamics were not biased by initial conditions when fitting to the observed case data starting in 2007.

VL case counts in small geographic units often exhibit high noise and variability, making it difficult to discern meaningful patterns. Therefore, we aggregated data from the municipality to the state level in Maranhão, reducing random fluctuations while preserving underlying trends. Importantly, climate drivers like ENSO influence temperature and precipitation across broad spatial scales, making their effects more consistently detectable at state or regional levels compared to finer geographic resolutions, where local variability can mask these large-scale signals (Hsiang & Meng, 2015; Rodo et al., 2002; Ropelewski & Halpert, 1987).

### 3.2 Simulated potential public health interventions

We assessed the effect of various potential interventions selected from existing strategies used in Brazil's national VL control program (Table S1), including increased vector mortality, canine euthanasia, canine treatment, and sandfly maturation rates, by simulating human cases after reducing or increasing the corresponding model parameters We increased sandfly mortality (*μ*v), increased canine mortality (*μ*_*r*_), reduced vector development (*mr*_*v*_), and increased clinical treatment rates among the infected canine reservoir population (tr_*d*_) by 10 %, 20 %, 50 %, and 90 %, considering each intervention separately. Therefore, each estimate and corresponding 95 % CI represent the simulated number of cases under hypothetical scenarios where the coverage or the effectiveness of each intervention was increased while all other factors were held constant.

We further evaluated the impacts of combined interventions using an analogous approach across multiple intervention strategies simultaneously. This combined strategy involves simultaneously increasing vector mortality, reducing sandfly maturation rate, and relies on non-lethal interventions for the canine reservoir (increasing canine treatment). Each intervention scenario was simulated across 1000 bootstrap iterations to calculate the median (point estimate) and 95 % confidence intervals (CI, 2.5th and 97.5th percentile of the bootstrap sample distribution) for potential reductions in cases from each intervention. Total case counts over the 13-year study period were compared to simulated and observed baseline case counts to understand how interventions might have decreased case counts during this period.

## 4 Results

**Fig. 2:**
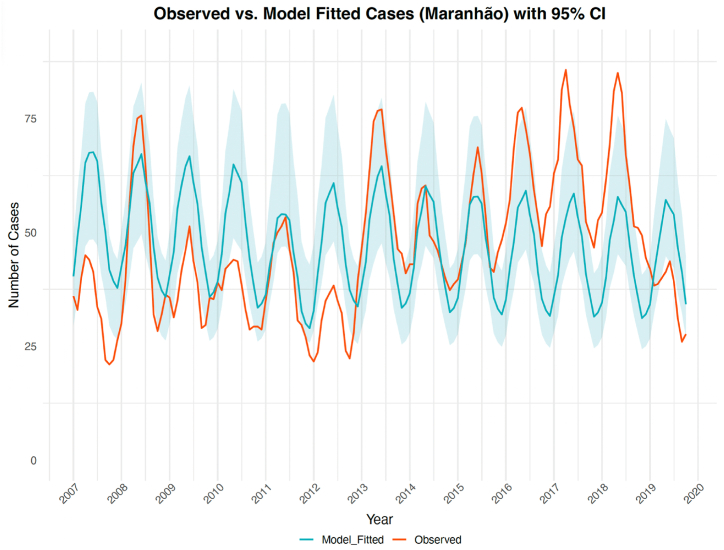
Observed versus model-predicted cases. The 95 % confidence interval derived from the 2.5th and 97.5th percentiles of the bootstrapped distribution is shaded in blue.

The model effectively captured the overall temporal trends and seasonality of observed case counts (Fig. 2). While the model underestimated the magnitude of several peak outbreaks, emphasizing the challenges in capturing extreme fluctuations, the simulated total number of cases from 2007 to 2019 (7280.43 cases (95 % CI: 5601.81–9350.32)) closely reflected the observed total cases (7,245).

**Fig. 3:**
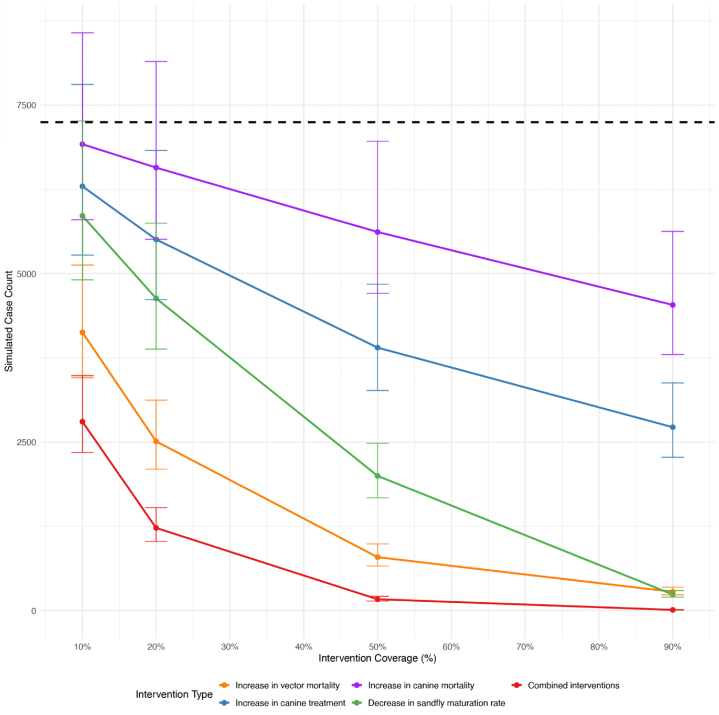
Intervention impacts on simulated VL case counts with 10 %, 20 %, 50 %, and 90 % simulated coverage for each intervention type. The black dashed line represents the total number of simulated cases at baseline (no intervention increase).

Our transmission model (Fig. 1) served as the baseline framework for evaluating counterfactual intervention simulations (Fig. 3).

In these simulated interventions, vector control was the most effective intervention for reducing VL cases. A 10 % increase in vector mortality resulted in a 43 % (95 % CI: 29.2–52.3 %) reduction in cases, demonstrating a significant impact on reducing cases even at lower levels of control (Table 2). As vector control efforts intensified, reductions in transmission became even more pronounced. A 90 % increase in sandfly mortality resulted in an 96.1 % (95.2–96.8 %) decrease in cases, with a total reduction of 7006 cases (95 % CI: 6897–7045).

**Table 2:** Estimated percent reduction in simulated visceral leishmaniasis cases compared to baseline across four potential intervention strategies. Each cell represents the modeled percent reduction in cases and associated 95 % confidence intervals at increasing intervention intensities (10 %, 20 %, 50 %, and 90 %).

% Change	Decrease in sandfly maturation rate	Increase in canine mortality	Increase in canine treatment	Increase in vector mortality
10	19.2 % (0.2–32.3 %)	4.5 % (−18.3–19.9 %)	13.1 % (−7.7-27.2 %)	43.0 % (29.2–52.3 %)
20	36.1 % (20.7–46.5 %)	9.3 % (−12.4–23.9 %)	24.0 % (5.8–36.3 %)	65.4 % (56.9–71.0 %)
50	72.4 % (65.7–76.9 %)	22.5 % (3.9–35.0 %)	46.1 % (33.2–54.9 %)	89.1 % (86.3–90.9 %)
90	96.7 % (95.9–97.2 %)	37.4 % (22.4–47.6 %)	62.5 % (53.4–68.6 %)	96.1 % (95.2–96.8 %)

We quantified the potential impact of environmental management interventions by reducing sandfly maturation rates, which represent the availability of breeding and development sites that rely on organic matter. Specifically, a 10 % reduction in sandfly maturation led to a 19.2 % (0.2–32.3 %) decline in human cases, while more aggressive interventions showed even greater benefits.

While canine treatment modestly reduced human cases, its impact remained significantly lower than vector control interventions. A 10 % increase in canine treatment resulted in a relatively small reduction of 950 cases. Even at a 90 % increase in treatment, case reductions only reached 4525 fewer cases (a 62.5 % reduction compared to the baseline).

Canine euthanasia has been a controversial control strategy in Brazil, often implemented to reduce transmission from infected dogs. We observed no substantial reduction in VL cases until canine mortality increased by up to 50 %, at which point cases were reduced by only 22.5 % (3.9–35 %). Even with a 90 % increase in canine mortality, transmission declined by just 37.4 % (22.4–47.6 %).

The combined intervention strategy resulted in a markedly steeper decline in simulated VL cases compared to any single intervention alone. Even at a 10 % increase in intensity, the combined approach led to a 61.3 % reduction in cases, and by 90 % coverage, case counts dropped by 99.8 % (total cases with combined approach: 11 (9, 13)), which is far lower than any individual strategy could achieve on its own (Fig. 3). This result highlights the synergistic potential of integrated approaches, where moderate gains from each component intervention accumulate to produce a substantial overall impact. In other words, a modest increase across multiple interventions can be more effective than a large increase in just one, underscoring the value of coordinated, multi-pronged strategies. Notably, the combined strategy relies on non-lethal interventions for the canine reservoir. While euthanasia has been widely used in endemic regions, our findings and those from other studies suggest that prioritizing treatment over removal may be equally or more effective, particularly when implemented alongside robust vector control measures.

## 5 Discussion

Our climate-driven model, which incorporates seasonal variations in sandfly biting rate and vector recruitment, and ENSO-dependent vector traits, provides new insights into VL dynamics in Maranhão, Brazil. The model successfully captured seasonal transmission patterns with the integration of ENSO variability. Our findings suggested that climate fluctuations, particularly ENSO-related variability, contribute to transmission dynamics in this region, influencing sandfly activity and transmission risk over time.

Although the ENSO-based model generally captured seasonal patterns in VL incidence, it underestimated some of the sharp epidemic spikes. These discrepancies are not unexpected, given the complex, multifactorial nature of VL transmission and the fact that ENSO alone cannot account for short-term or localized drivers of outbreak dynamics. ENSO likely influences VL through a cascade of ecological changes such as shifts in rainfall, humidity, and vector abundance that may operate over longer or cumulative time scales. Additionally, unmeasured factors such as local outbreaks, health system disruptions, or changes in surveillance efforts and vector control efforts could contribute to changes in case counts that cannot be captured by our model. To improve the model performance, incorporating more flexible lag structures, additional environmental and non-environmental covariates, and context-specific drivers, particularly during periods of climatic extremes, may be needed. Still, our model was able to capture the overall trend of VL cases in this area, highlighting its utility as a framework for testing potential intervention scenarios and exploring climate-sensitive transmission dynamics, accounting for the complex interplay of humans, reservoirs, and vectors.

Our results showed that no single intervention could be expected to fully eliminate VL transmission, but among the potential interventions tested, vector control remained the most effective strategy. In our model, a 10 % increase in sandfly mortality, used as a proxy for modest, achievable improvements in adult vector suppression, led to a 43 % reduction in human cases. While we referred to this increase as “modest,” it reflected a relatively small perturbation in the vector mortality rate compared to baseline values used in entomological studies and was intended to represent incremental gains from strengthening existing insecticide programs. At the upper end, a 90 % increase in vector mortality resulted in a 96 % decrease in cases; while this level is likely practically implausible under field conditions, it served to illustrate the hypothetical maximum impact of aggressive vector suppression.

Importantly, achieving substantial sandfly mortality in practice is challenging in Brazil due to a combination of biological, operational, and structural constraints. These may include the proliferation of vectors in increasingly urban and peri-urban environments, where breeding sites are widespread and difficult to target; decentralization of vector control activities, which has led to uneven municipal capacity; high operational costs associated with sustained insecticide application; and growing operational barriers. While current control programs primarily focus on adult sandflies, which can be particularly difficult to target, our results suggest that environmental management strategies that reduce sandfly breeding sites offer a promising approach to VL control in Maranhão.

VL transmission risk is closely linked to poor sanitary conditions, leaf litter accumulation, and the presence of domestic animals, including dogs, chickens, and livestock (Vianna et al., 2016) Our model suggests that reducing sandfly maturation rates, which can be achieved through interventions such as improved sanitation, habitat modification, and organic matter removal, could substantially lower the transmission. Specifically, we found that a 10 % reduction in sandfly maturation led to a 19 % decrease in human cases, while a 50 % reduction lowered cases by 72 %, and a 90 % reduction resulted in a 97 % decline in human cases. These findings underscore the potential of environmental management as a sustainable and climate-resilient strategy, particularly in underserved areas where sanitation services and waste management remain inadequate. This approach may be especially effective given that sandflies undergo a prolonged developmental phase, lasting over 30 days, during which they remain largely immobile, making larval-stage interventions a promising avenue for targeted control (da Saúde www. saude. gov. br/bvs, n.d.). Maranhão is among the poorest states in Brazil, with low household income, limited sanitation infrastructure, and low educational attainment. These structural determinants hinder the feasibility of environmental management interventions such as routine waste removal and improved sanitation. Our estimates therefore reflect biological potential rather than guaranteed real-world effectiveness in resource-constrained settings.

Interestingly, we observed a crossover between the estimated impacts of increasing vector mortality and decreasing sandfly maturation rate (Fig. 3). Increasing vector mortality led to an immediate decline in VL cases at lower intervention intensities, but its marginal effectiveness diminished at higher levels as most transmissible vectors were already being removed. In contrast, reducing the sandfly maturation rate resulted in a slower initial effect but achieved greater long-term reductions in transmission at high intervention levels by limiting the emergence of new adult vectors and suppressing population regeneration more sustainably. These differing patterns likely explained the crossover and highlighted the value of transmission models in assessing intervention impacts by capturing complex, non-linear dynamics.

Since domestic dogs serve as a key reservoir for VL transmission, we evaluated the impact of expanded canine treatment on case reduction. Our findings indicated that canine treatment alone provided only moderate benefits. These results suggested that canine treatment should be considered a complementary rather than primary intervention, highlighting the potential for approaches that integrate reservoir management with vector suppression and environmental interventions. Treatment and clinical cure do not always result in a parasitological cure among infected dogs, meaning that they may remain infectious to sandflies.

Canine euthanasia has been widely used in Brazil but remains controversial due to ethical concerns, limited public acceptance, and operational challenges. In our model, increasing canine mortality alone resulted in minimal impact on VL transmission, with no meaningful reductions in case counts until mortality was increased by 50 %, at which point cases declined by only 23 %. These results align with previous studies suggesting that culling is not an effective standalone strategy in zoonotic VL settings (Vaz et al., 2020). An alternative reservoir-targeted intervention not included in our model is canine vaccination. Although not currently implemented at scale in Brazil, vaccination has been proposed as a more sustainable and socially acceptable strategy for reducing canine infectiousness. However, evidence on vaccine efficacy and cost-effectiveness remains limited, and there is currently insufficient data to confirm that vaccination reduces transmission from infected, vaccinated dogs to sandflies to a level that would meaningfully lower the risk of *L. infantum* infection or VL in humans (Dantas-Torres et al., 2020). As such, while canine vaccination may hold future promise, its role in public health–oriented VL control programs requires further investigation through field trials and transmission studies.

Other interventions, such as insecticide-treated nets, may also improve personal protection against infectious bites. Additionally, nets may contribute to reducing the transmission of other vector-borne diseases where humans serve as primary or amplifying hosts. However, in the case of zoonotic diseases like VL, where humans are incidental hosts and do not contribute meaningfully to ongoing transmission, the use of nets alone is unlikely to substantially interrupt the broader transmission cycle. Therefore, while nets may offer individual-level protection, their population-level impact on VL control is expected to be limited without complementary measures targeting the sandfly vector or animal reservoirs.

While our model effectively captured seasonal and climate-driven VL transmission patterns in Maranhão, several limitations should be acknowledged. First, although ENSO variability improved model performance, ENSO's influence on transmission is indirect and mediated through factors such as temperature, humidity, and rainfall. Future studies should explore multi-climate variable models that explicitly incorporate temperature-dependent sandfly survival, breeding, and biting behavior. For this to be most effective, further laboratory studies are needed to evaluate *Lutzomyia longipalpis* behaviors at different temperatures, particularly since this vector does not tend to behave in the same manner as other, more extensively studied disease vectors, such as *Anopheles* or *Aedes* mosquitoes. Second, our model did not account for human mobility or long-term changes in vector distribution due to land use shifts or climate change. These factors may contribute to transmission patterns in ways not fully captured by the current framework. Third, although VL incidence has declined in some regions of Brazil in recent years, interpreting trends after 2020 is challenging due to substantial COVID-related reporting disruptions. Future analyses should incorporate these years once robust reporting completeness estimates or correction methods become available. Finally, geographic scaling was necessary to reduce noise in case counts. In smaller administrative regions, relatively low VL incidence can lead to high variability in reported cases, making it difficult to extract clear seasonal trends. To address this, we aggregated data from municipalities to the state of Maranhão, which improved signal detection for seasonal fluctuations but may have masked fine-scale heterogeneity in local transmission dynamics.Although our analysis focuses on ENSO as a large-scale climate driver, it is important to situate these findings within the broader context of climate change. Long-term warming, altered rainfall regimes, and intensifying climatic extremes may influence sandfly survival, breeding habitats, and human exposure patterns in ways that amplify or attenuate the ENSO-linked mechanisms evaluated here. These long-term climatic shifts could modify baseline sandfly abundance or extend the seasonal window of transmission. While projecting such future changes is beyond the scope of this study, our framework provides a foundation that could be coupled with climate-projection scenarios in future work.

This study provides a framework for understanding how ENSO variability and seasonality influence VL transmission dynamics and for evaluating the potential impact of targeted interventions. This work underscores the importance of interdisciplinary collaboration across entomology, veterinary science, epidemiology, and community-based research to inform integrated control strategies. While vector control remains a critical intervention, long-term VL management will require coordinated efforts that also address reservoir hosts and ensure equitable access to diagnosis and treatment.

## 6 Conclusion

Our findings reinforce that vector control and environmental management are the most effective interventions for reducing VL transmission in Maranhão. The incorporation of ENSO-dependent transmission highlights the role of climate variability in shaping transmission patterns, suggesting that climate-informed public health responses may improve control efforts. While canine treatment provides moderate benefits, it is insufficient as a standalone strategy, and canine culling remains largely ineffective.

## CRediT authorship contribution statement

**Quinn H. Adams:** Writing – original draft, Visualization, Methodology, Investigation, Formal analysis, Data curation, Conceptualization. **Davidson H. Hamer:** Writing – review & editing, Supervision, Conceptualization. **Lucy R. Hutyra:** Writing – review & editing, Conceptualization. **Gregory A. Wellenius:** Writing – review & editing, Supervision, Investigation, Funding acquisition, Formal analysis, Conceptualization. **Kayoko Shioda:** Writing – review & editing, Validation, Supervision, Methodology, Formal analysis, Data curation, Conceptualization.

## Financial Disclosure statement

QHA is supported by a T32 training grant awarded to the Department of Environmental Health at the Boston University School of Public Health (T32ES014562), a National Science Foundation Research Traineeship (NRT) grant to Boston University (DGE 1735087), and funds from an unrestricted gift previously made by Google.org to Boston University.

## Declaration of competing interest

The authors declare the following financial interests/personal relationships which may be considered as potential competing interests:Dr. Wellenius serves as a consultant for the Health Effects Institute (Boston, MA) and Google, LLC (Mountain View, CA).
