# Supplementary material for: Modeling the seasonal and climate-dependent dynamics of visceral leishmaniasis in Brazil: Implications for transmission and Control
Source: Infect Dis Model. 2025 Nov 25;11(2):549–59. doi: 10.1016/j.idm.2025.11.009 (PMC12757486; doi:10.1016/j.idm.2025.11.009)
Supplement: Multimedia component 1 [file mmc1.docx]

Modeling the Seasonal and Climate-Dependent Dynamics of Visceral Leishmaniasis in Brazil: Implications for Transmission and Control

Supplemental Materials

Quinn H. Adams^1,2^, Davidson H. Hamer^3,4,5^, Lucy R. Hutyra^6^, Gregory A. Wellenius^1,2^, Kayoko Shioda^3,4^

1. Center for Climate and Health, Boston University School of Public Health, Boston, Massachusetts, United States of America
2. Department of Environmental Health, Boston University School of Public Health, Boston, Massachusetts, United States of America
3. Department of Global Health, Boston University School of Public Health, Boston, Massachusetts, United States of America
4. Center on Emerging Infectious Diseases, Boston University, Boston, Massachusetts, United States of America
5. Section of Infectious Diseases, Department of Medicine, Boston University Chobanian & Avedisian School of Medicine
6. Department of Earth and Environment, Boston University, Boston, Massachusetts, United States of America

Table S1: *Overview of existing visceral leishmaniasis (VL) control measures implemented in Brazil’s national VL control program, categorized by intervention type. Strategies are grouped into four categories: vector control, environmental management (targeting sandfly maturation), expanded canine treatment, and increased canine mortality.*

| **Category** | **Control Measure** | **Description** |
| --- | --- | --- |
| **Vector Control** | Indoor Residual Spraying (IRS) | Application of insecticides (e.g., pyrethroids) on interior walls of homes and animal shelters to eliminate adult sandflies. |
|  | Use of Insecticide-Treated Dog Collars | Collars impregnated with insecticides (e.g., deltamethrin) to protect dogs from sandfly bites, thereby reducing transmission. |
|  | Personal Protective Measures | Use of bed nets, repellents, and protective clothing to prevent sandfly bites, especially during peak activity periods. |
| **Environmental Management** | Sanitation of Peridomestic Areas | Regular cleaning of yards and animal shelters to remove organic matter that serves as breeding sites for sandflies. |
|  | Proper Waste Disposal | Ensuring appropriate disposal of organic waste to reduce sandfly breeding grounds. |
|  | Vegetation Management | Pruning trees and clearing vegetation near homes to decrease humidity and shade, making the environment less favorable for sandflies. |
| **Expanded Canine Treatment** | Serological Surveys and Treatment | Conducting regular serological testing of dogs and providing treatment to infected animals to reduce reservoir potential. |
|  | Vaccination | Vaccines (e.g., Leish-Tec) are available for individual canine protection. Note: Canine vaccination is not formally included in Brazil’s national VL control program due to insufficient evidence of population-level impact. |
| **Increased Canine Mortality** | Euthanasia of Infected Dogs | Identification and euthanasia of seropositive dogs to eliminate reservoirs of the parasite. |
|  | Control of Stray Dog Populations | Implementing measures to manage and reduce stray dog populations, which can serve as reservoirs for VL. |
